# INSL3 Variation in Dogs Following Suppression and Recovery of the HPG Axis

**DOI:** 10.3390/ani14050675

**Published:** 2024-02-21

**Authors:** Ravinder Anand-Ivell, Acacia Rebello Coutinho, Yanzhenzi Dai, Gary England, Sandra Goericke-Pesch, Richard Ivell

**Affiliations:** 1 School of Bioscience, University of Nottingham, Sutton Bonington Campus, Sutton Bonington LE12 5RD, UK; 2 School of Veterinary Medicine and Science, University of Nottingham, Sutton Bonington Campus, Sutton Bonington LE12 5RD, UK; 3 Unit for Reproductive Medicine, Clinic for Small Animals, University of Veterinary Medicine Hannover, 30559 Hannover, Germany

**Keywords:** INSL3, male contraception, GnRH implant, HPG axis, canine

## Abstract

**Simple Summary:**

Insulin-like peptide 3 (INSL3) is a hormone produced by the steroidogenic Leydig cells of the testes and in most mammals acts as a robust blood biomarker for pubertal development and gonadal function. Using a new immunoassay to measure INSL3 in adult male dogs, we show that it is much lower than in other species. When a cohort of male beagle dogs is implanted with a GnRH agonist to induce contraceptive suppression of male fertility, the average circulating INSL3 is first reduced and then recovers slowly when the implant is removed. Importantly, however, and unlike in other species, in dogs there is considerable variation in INSL3 expression, with some dogs showing little or no initial reduction of INSL3, and similarly, there is often only a poor response during the recovery phase. We conclude that in dogs, unlike in most other mammals, the Leydig-cell production of INSL3 appears to be less tightly coupled to the functioning of the hypothalamic–pituitary–gonadal (HPG) hormone axis.

**Abstract:**

Insulin-like peptide 3 (INSL3) is a constitutive product of mature, adult-type Leydig cells of the testes and consequently in most mammals is an ideal biomarker with which to monitor pubertal development. A new heterologous time-resolved fluorescence immunoassay was developed and validated to measure circulating INSL3 in the blood of adult male dogs. Compared to other species, INSL3 concentration is low with marked variation between individuals, which appears to be independent of breed, age, or weight. A model system was then used in which a cohort of beagle dogs was subject to a GnRH-agonist implant to suppress the HPG axis and spermatogenesis, followed by implant removal and recovery. Unlike testosterone, INSL3 levels were not fully suppressed in all animals by the GnRH agonist, nor was the recovery of Leydig cell function following implant removal uniform or complete, even after several weeks. In dogs, and dissimilar from other species (including humans), Leydig-cell INSL3 appears to be quite variable between individual dogs and only weakly connected to the physiology of the HPG axis after its suppression by a GnRH-agonist implant and recovery. Consequently, INSL3 may be less useful in this species for the assessment of testis function.

## 1. Introduction

In the male mammal, the Leydig cells of the testes in the foetus and in the adult are the principal sources of testosterone, essential for the masculinisation of the male phenotype and for the maintenance of adult male characteristics and physiology. A disruption of testosterone synthesis or secretion leads to hypogonadism, which in the adult human male is significantly associated with loss of sexual function and behaviour, cognitive decline, symptoms of metabolic syndrome, bone loss and muscle weakness [1]. There are two main populations of Leydig cells: the first develops during early pregnancy in the male foetus to provide testosterone for foetal masculinisation. This population appears to involute around birth so that in infancy there is no circulating testosterone. Puberty is initiated with the activation of the hypothalamic–pituitary–gonadal (HPG) axis somewhat later, depending on the species. Pulsatile GnRH from the hypothalamus stimulates gonadotropes in the anterior pituitary to produce the phasic gonadotropins, luteinizing hormone (LH) and follicle-stimulating hormone (FSH). These, in turn, react with specific receptors on testicular Leydig and Sertoli cells, respectively, to initiate cell differentiation and proliferation, thereby causing the induction of steroidogenesis and spermatogenesis, which are the hallmarks of pubertal development. In the adult male, testosterone production remains acutely regulated by LH from the HPG axis, where it is instrumental via negative feedback to the hypothalamus and pituitary gland in maintaining sufficient circulating testosterone to support adult male physiology, also including spermatogenesis.

The Leydig cells of the testes also produce a second circulating hormone: insulin-like peptide 3 (INSL3) [2]. This hormone is essential to promote the first phase of testicular descent in the foetus, where it directs the development and shortening of the gubernacular ligament connecting the testes to the inguinal region and preparing this for eversion of the testes into the scrotum. INSL3 acts via a unique G-protein-coupled receptor on target cells, called RXFP2 (relaxin family peptide receptor 2). In the adult, Leydig cells also produce INSL3, which is involved in promoting spermatogenesis (pigs, rodents, humans [3,4,5]) and in supporting bone and skeletal muscle metabolism (humans, rodents [6,7]). Moreover, in ruminants, the INSL3-RXFP2 hormone-receptor system is functionally linked to horn growth and status [8,9]. Unlike testosterone, circulating INSL3 is not acutely regulated by the HPG axis but is nevertheless chronically modulated by LH, or lack of it, through the role of LH in inducing the differentiation of mature Leydig cells from less-differentiated precursors or stem cells [10]. Consequently, within adult men with a stable HPG axis, INSL3 levels remain remarkably constant, reflecting only the number and differentiation status of the Leydig cells, and hence their functional capacity [11]. Within individuals, INSL3 concentration declines gradually with age, reflecting the slow decline in Leydig-cell functional capacity and/or numbers in middle-aged and older men [12,13]. There is, however, high between-individual variation in INSL3 levels in men, of which the origin is still not understood.

Circulating INSL3 has been studied in a range of mammal species, mostly varying in young adult males between 0.5 and 30 ng/mL (Table 1). Dogs appear to be an exception, with only very low INSL3 concentrations reported [14,15]. 

The present investigation was undertaken firstly to validate the putatively low concentration of circulating INSL3 in different breeds of dog, and secondly, to determine whether a reduced expression of INSL3 implies an altered relationship with the hormones of the HPG axis and hence with male physiology. For this purpose, we have made retrospective use of a large and well-documented historical cohort of beagle dogs subjected to a contraceptive regimen of a GnRH-agonist implant. The uniformity of the dogs, the suppression of the HPG axis, and its synchronized recovery phase, in some respects effectively replicating Leydig cell differentiation during puberty, allow detailed insight into the interactions between INSL3 expression, Leydig cell function, and the physiology of the HPG axis. 

## 2. Materials and Methods

### 2.1. Validation of a Heterologous Immunoassay to Measure Canine INSL3

From an evolutionary perspective, dogs are more closely related to ruminants than to primates or rodents [27]. For this reason, a well-established time-resolved fluorescent immunoassay designed to measure bovine INSL3 [28] was adapted and validated to assess canine INSL3 in dog blood plasma or serum. A similar approach has also been used by Pathirana and colleagues [14]. The assay essentially followed the same format as previously [28] using Eu^3+^-labelled bovine INSL3 as a tracer and the same anti-bovine INSL3 primary polyclonal antibody raised in rabbits. Titration of blood from male foetal calves and male dogs showed complete parallelism (Supplementary Figure S1A). Although bovine INSL3 was used as a routine internal standard, independent validation using synthetic canine INSL3 (a generous gift from Professors N. Kawate and E.E. Bullesbach) showed identical values. The working range of the assay was 0.02 to 10 ng/mL INSL3 (Supplementary Figure S1B), with a lower limit of quantification (LOQ) of 0.05 ng/mL. For statistical purposes, values generated less than the LOQ were retained without alteration. The inter- and intra-assay coefficients of variation (COV) were both consistently <5%. Previous studies have shown that the original assay indicated no cross-reactivity with any related peptides, including human and porcine relaxin, human and bovine insulin, bovine IGF1 and IGF2, and human INSL6. 

### 2.2. Animals and Study Design

As part of the original characterization, discarded blood serum from 32 male dogs of various breeds and of a range of post-pubertal ages (5–12 years) and weights were made available. Discarded serum samples were also collected from four female dogs and five previously castrated adult male dogs. All dogs were clinically healthy at the time of sample collection, and they are referred to as ‘English’ dogs.

**Figure 1:**
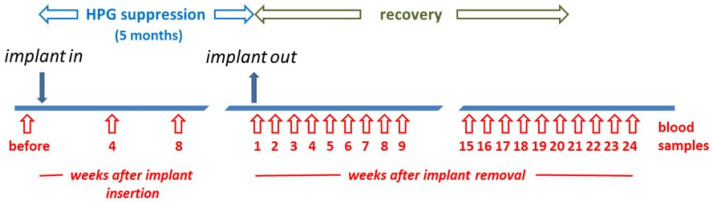
Scheme for the German cohort of Beagle dogs to indicate timing of Gonazon^®^ implant insertion and removal, as well as blood sampling strategy. The week in which the implant was removed is labelled week 1.

Secondly, archived blood (stored at −25 °C) was made available from a cohort of 59 adult male beagle dogs (aged 1–6 years), which are later referred to as ‘German’ beagles. Previous studies have shown that INSL3 is very stable over long periods when stored at −20 °C [2] (and unpublished). Thirty-five of these dogs had received a depot implant of a GnRH agonist (18.5 mg azagly-nafarelin; Gonazon^®^; Intervet Pharma, Angers Technologie, Beaucouzé, France) to suppress the HPG axis and hence spermatogenesis. As part of a previously reported, ethically approved research study, the implant was retained for 5 months and then removed, with blood sampling for each animal before implantation, 4 and 8 weeks after implantation, and then weekly after the removal of the implant at 5 months up to a maximum of 24 weeks (Figure 1). In addition, though not used for the present study, some animals were castrated, and the testes were subject to histology and RT-PCR analysis at intervals following removal of the Gonazon^®^ implant. This cohort has been the subject of several studies relating to testis function and endocrinology, with detailed descriptions already provided [29,30,31,32]. Some relevant results from these earlier studies are included in Supplementary Figure S2.

### 2.3. Statistics

All data were analysed using simple descriptive and column statistics employing GraphPad Prism (version 8.0). Significant differences (*p* < 0.05) were estimated using one-way ANOVA followed by Dunnett’s multiple comparison tests or, where appropriate, *t*-tests.

### 2.4. Ethical Approvals

All English blood samples from clinical cases were collected for routine clinical health monitoring as allowed under the UK Veterinary Surgeons Act; any blood that was collected that was in excess of that required for the clinical reason was used with the permission of the owner for the purposes of this study. For the study in beagles, animal experimentation was approved by the respective authority (permit No. AZ V54-19c20/15c GI18/14, Regierungspräsidium Giessen).

## 3. Results

### 3.1. Effect of Breed, Age, and Weight

**Figure 2:**
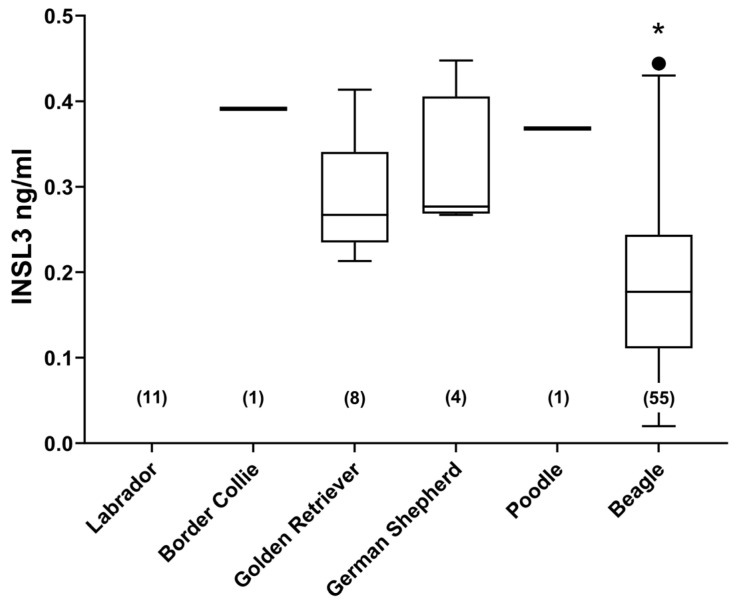
Circulating INSL3 concentration (mean ± 95% CI) in different breeds of dog as indicated. Only single samples were available for Border Collie and Poodle. N-values are indicated on the *x*-axis. Only Beagles were significantly different (*, *p* < 0.05) from any of the other breeds studied.

**Figure 3:**
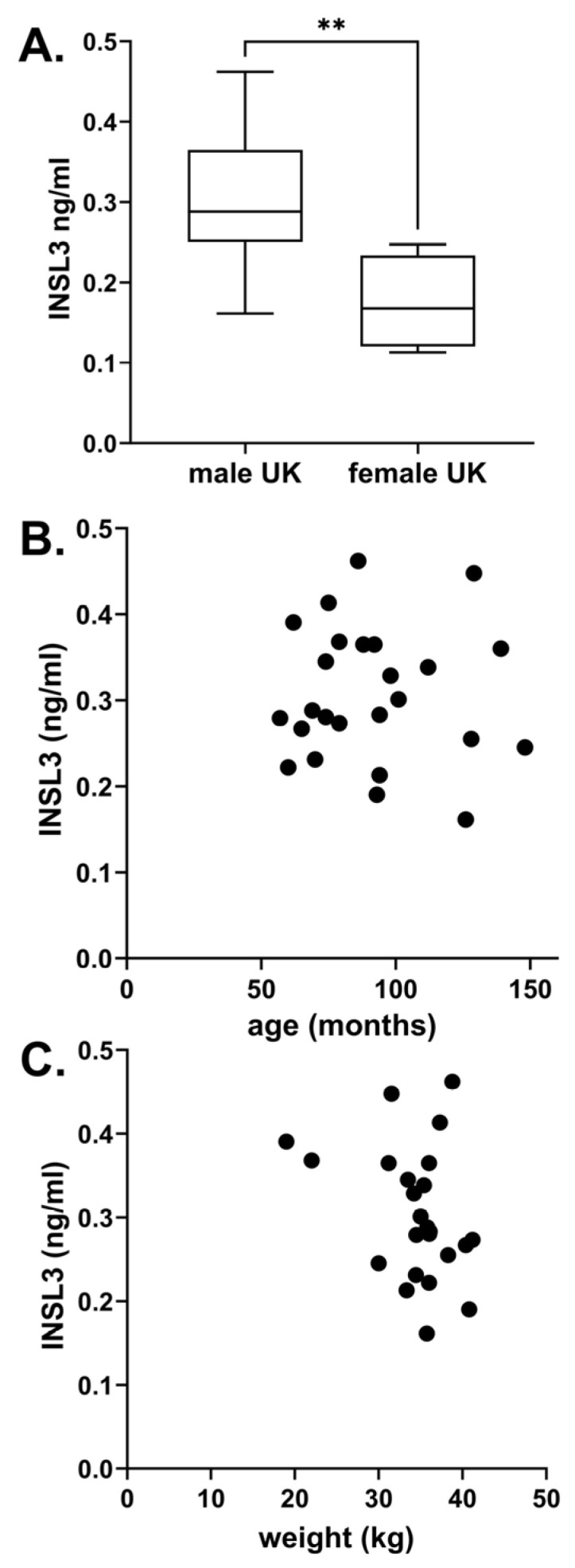
(**A**) Circulating INSL3 (mean ± 95% CI) in serum from English male dogs (excluding Beagles) compared to similarly aged females of equivalent breeds (**, *p* < 0.01). (**B**) Scatterplot of INSL3 vs. age and (**C**) Scatterplot of INSL3 vs. weight for all English male dogs. (**B**,**C**) indicated no significant relationship.

Altogether, six different breeds of dog were studied, including unimplanted controls from the cohort of beagles (Figure 2). There were no significant differences between any of the assessed English dog breeds. However, the German beagles indicated a mean INSL3 concentration significantly lower than the other breeds. This may have been due to the average younger age of these dogs, their overall smaller size, or greater uniformity. Of the non-beagle breeds, no significant relationship was identified between INSL3 concentration and body weight, nor with age (Figure 3). The four female dogs indicated significantly lower INSL3 concentrations compared to the male dogs of similar breeds (Figure 3A). The five castrated male dogs all indicated INSL3 concentrations below the LOQ (0.029 ± 0.009 ng/mL).

### 3.2. Effect of HPG Axis Suppression

**Figure 4:**
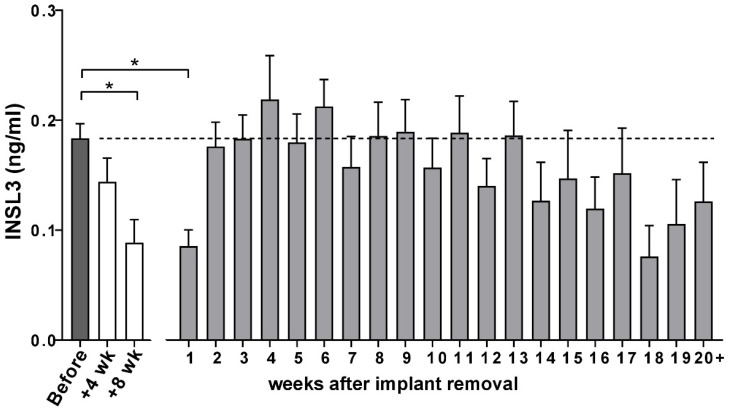
Circulating INSL3 (means + SEM) in the German cohort of male Beagle dogs at indicated times before and after insertion of the Gonazon^®^ implant and after its removal. Significant differences (*, *p* < 0.05) were determined by ANOVA followed by Dunnet’s test for multiple comparisons.

**Figure 5:**
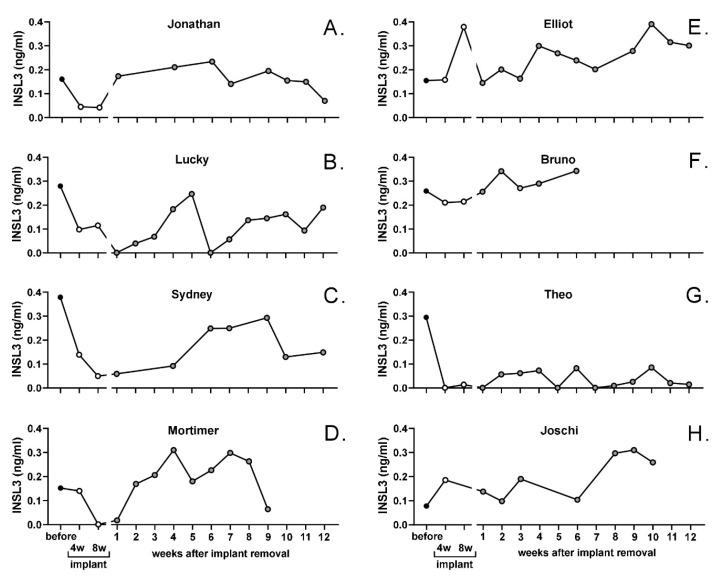
Profiles of circulating INSL3 (single values) in individual male dogs (panels (**A**–**H**)) selected from the German Beagle cohort to illustrate variation in response to the Gonazon^®^ implant and its removal. Data represented up to week 12 following implant removal.

For the whole cohort of beagles, circulating INSL3 concentration was significantly suppressed by the depot GnRH-agonist implant, though at 8 weeks this still did not indicate the complete suppression of mature Leydig cell function (Figure 4). This was largely due to heterogeneity between individual animals, with some animals indicating good suppression (e.g., Figure 5A,C,D,G) and others only having poor or no apparent Leydig cell suppression at this stage (e.g., Figure 5D,E,H).

### 3.3. Recovery of Leydig-Cell Functional Capacity Following Removal of the Gonazon^®^ Implant

For the entire beagle cohort, there was evident recovery of circulating INSL3 to pre-implantation levels already at 2–3 weeks, attaining a maximum at 4–5 weeks (Figure 4). Thereafter, there appeared to be a downward trend in circulating INSL3 levels, though trend analysis showed this not to be significant, probably due to the high between-individual variation in response. This variation is illustrated for selected individuals in Figure 5, which indicates good recovery in some animals (e.g., Figure 5A,C or Figure 5D) and either poor (e.g., Figure 5G) or sporadic recovery (e.g., Figure 5B) in others. These individuals were selected to show the range of profile variation encountered, including extreme cases. Dogs not illustrated showed profiles intermediate between those shown in Figure 5.

## 4. Discussion

Whereas all other mammalian species whose INSL3 has been measured in peripheral blood have healthy adult male concentrations ranging from 0.5 to 30 ng/mL (Table 1), dogs appear to be unusual in having much lower circulating post-pubertal concentrations (0.02–0.46 ng/mL, present study; 0.005–0.43 ng/mL [14,15]). Why this is so is unclear, though it may reflect an adult physiology largely dependent on paracrine rather than endocrine INSL3 functionality. It is of interest that the female dogs used as controls also indicated low but significant circulating INSL3 levels (Figure 3A). This has also been shown for young women of reproductive age [33] as well as in cows [28] and largely reflects local paracrine roles within the ovaries. This is also implied by the circulating values of INSL3 in the castrated dogs, which were all below the LOQ, suggesting no alternative non-gonadal sources of INSL3. Appropriate studies in female dogs are lacking. 

The application of an endocrine male contraceptive paradigm, here through the suppression of the HPG axis by use of a GnRH-agonist implant in young adult animals, is particularly informative. Not only does such a paradigm provide information as to the extent to which spermatogenesis and/or steroidogenesis is dependent on a functional HPG axis, but the recovery phase following the removal of the implant is considered to reflect those developmental and differentiation steps that otherwise only occur during puberty or in seasonal breeders. Something similar also occurs, for example, during HPG axis suppression induced by anabolic steroid misuse [34].

In the present retrospective study, it is evident that the GnRH agonist fulfilled its purpose of suppressing the production by the anterior pituitary of both gonadotropins, FSH and LH, to very low levels (Supplementary Figure S2), with a concomitant, almost complete suppression of spermatogenesis [29], as well as of testosterone production by the Leydig cells (Supplementary Figure S2). After implant removal, the HPG axis was restored and circulating levels of FSH, LH, and testosterone attained similar values to those prior to implant insertion. This occurs after 3 weeks for FSH, 6 weeks for LH, and 8 weeks for testosterone, reflecting the approximate timescale for re-establishing appropriate endocrine feedback criteria for the HPG axis, and similar to the expected dynamics in puberty. Full spermatogenesis required a longer time, with elongated spermatids first evident only after 9 to 24 weeks (mean 16.9 weeks [29]).

INSL3 expression appears to follow a less predictable pattern. Firstly, unlike the hormones of the HPG axis, INSL3 is not suppressed by the GnRH-agonist implant to very low circulating levels, except in a few dogs only. In other mammals, such as rodents or humans [11], INSL3 synthesis and secretion is a biomarker of mature Leydig cells, which have attained advanced differentiation status. For this reason, it is an excellent biochemical marker for pubertal development in most male mammals studied [8]. Whereas the proliferation and differentiation of early-stage Leydig cells are under LH control, the acute production of INSL3 is not, unlike testosterone [35]. In the adult male, INSL3 is effectively constitutive, reflecting only the numbers and/or differentiation status of the Leydig cells. Therefore, in seasonal breeders [11,36], or in men subject to a steroidal contraceptive regimen [37], reduced INSL3 reflects the loss and/or dedifferentiation of the mature Leydig cells. For example, Amory et al. [4] showed that circulating INSL3 was reduced from ca. 0.8 ng/mL to less than 0.1 ng/mL in men receiving steroidal contraception. Very similar results were obtained for men receiving a GnRH agonist as therapy for prostate cancer or in the treatment of MF transgender subjects [37]). In these examples, suppression of the HPG axis leads to an almost complete loss of INSL3 expression by the Leydig cells.

Secondly, in the recovery phase in dogs, the pattern of INSL3 is not consistent. In this phase, elevated LH should be encouraging the re-differentiation of Leydig cells and regeneration of new Leydig cells from resident precursor and stem cells. Although the average INSL3 concentration regains pre-implant levels at 4 weeks, individual profiles exhibit very high heterogeneity, suggesting that individual animals or their Leydig cells may not be uniform in their responsiveness to the gonadotropin. Using an immunohistochemical approach in the same cohort, Gentil et al. [32] suggested that the “upregulation of protein synthesis in individual Leydig cells occurs at a faster rate than the reactivation of resting cells”. Adult men also show high between-individual variance in circulating INSL3 concentration [13]. Although few studies are available for comparison, it is notable that in young men (mean age 24 years) receiving a steroidal contraceptive regimen, circulating INSL3 recovers to pre-treatment levels [37], whereas in somewhat older individuals (mean age 34 ± 8 years) recovering from anabolic steroid application, circulating INSL3 in long-term recovery remains significantly below control levels [34]. We have also shown that in middle-aged and older men with so-called “compensated” hypogonadism, where elevated gonadotropins have promoted testosterone production to normal eugonadal levels [38], circulating INSL3 still remains significantly reduced [39]. Taken together, such observations suggest that the full recovery of Leydig-cell functional capacity in men following the reinstatement of the HPG axis may be age-dependent, with younger individuals better able to respond to gonadotropins than those older. 

In beagles, the first motile sperm are evident in ejaculates at ca. 8–9 months [39], with maximum sperm concentration at 10–11 months [40], similar to the final fusion of bone ossification centres [41]. Thus, age does not appear to be a key factor in the beagle cohort since the majority of animals were aged 12–15 months (i.e., after the stable establishment of the HPG axis in this breed). Moreover, in the few (n = 5) older dogs (aged 5–6 years), the profile pattern was similarly diverse, with no evident impact of age. However, this may imply that the regulation of INSL3 expression in male dogs is less tightly controlled than in other species, unlike for testosterone. It is possible that domestication and artificial selection for non-survival traits have allowed a relaxation of selection pressure on Leydig cell development. This might also account for the unusually low and variable circulating concentration of INSL3 in adult dogs. A similarly diverse response to FSH was also observed in these dogs during the recovery phase regarding spermatogenesis, with considerable variation evident in the timing of germ-cell differentiation [29]. In a recent study of Bernese Mountain dogs [15], there was similarly no relationship evident between INSL3 concentration and age, weight, or seminal parameters.

Histological assessment of the testes from the beagle cohort at the completion of the original study showed that none of the dogs had signs of tumours or larger lesions [28,30], except for one (excluded from the present investigation), even though treatment with the slow-release GnRH-agonist implant induced an arrest of spermatogenesis at the level of spermatogonia/spermatocytes [29]. Even if no major/large “pathological” abnormalities were identified in the testes, it cannot be completely excluded that small local lesions might have existed, since the evaluation of the entire testes was not practicable. Additionally, it is well known that, even in healthy animals with physiological endocrine parameters, histopathological deviations (local Sertoli cells only, local arrests of spermatogenesis) can be observed [42]. In summary, it appears unlikely that aberrant histopathology could account for the variation in INSL3 profiles.

Furthermore, although male dogs are not considered seasonal in their testicular status, they are evidently responsive to some environmental cues, such as temperature, photoperiod, or the presence of female pheromones, leading to a large temporal variance in reproductive behaviour [43,44]. Such individual variance is also evident in the uncertainty about determining pubertal dynamics [44]. Future studies will need to assess the extent to which INSL3 expression could be useful as an additional parameter to monitor such development.

## 5. Conclusions

A well-validated, robust, specific, and sensitive heterologous immunoassay was developed to measure INSL3 in adult male dogs. Applied to male dogs receiving a GnRH-agonist implant to ensure the contraceptive suppression of spermatogenesis, followed by recovery after implant removal, the immunoassay revealed that unlike the hormones of the HPG axis, INSL3 in this species appears to be less tightly coupled to HPG axis physiology, possibly reflecting domestic canine evolutionary history. As a result, INSL3 may not be as significant a biochemical parameter for the assessment of testicular function as in other species.

## Figures and Tables

**Table 1:** Estimated circulating concentrations of INSL3 in various mammal species.

Species		Mean ± SD/Range (ng/mL)	References
Rat	(Sprague Dawley)	2.8 ± 0.2	[16]
	(Wistar)	1.5 + 0.1	[16]
Mouse		0.78 + 0.03	[16]
Human		1.3 ± 0.5	[12]
		1.8 ± 1.1	[17]
		1.3 (95%CI 0.9–2.7)	[18]
		0.94 ± 0.31	[19]
Macaque		0.44 ± 0.13	[20]
Goat		10–30	[21]
Bull		10–20	[22]
		3–6	[23]
		6-10	[24]
Pig		10–15	[25]
Horse		19.9 ± 17.7	[26]
Dog		0.05–0.43	[14]
		0.09 (0.005–0.163) ^a^	[15]

^a^ median and interquartile range.

## Data Availability

All data is presented within the manuscript or Supplementary Materials, or can be made available upon reasonable request from the corresponding author.

## Supplementary Materials

The following supporting information can be downloaded at: https://www.mdpi.com/article/10.3390/ani14050675/s1, Figure S1: A. Serial dilution of sera from two fetal calves and two male dogs, to indicate assay parallelism. B. Titration curve for the Eu^3+^-labelled tracer against pure canine INSL3 using the heterologous bovine INSL3 assay; Figure S2: Hormone profiles measured in blood samples collected at the indicated times from the cohort of German male Beagle dogs. A. Testosterone. B. LH. C. FSH. For details see [28].
