# Supplementary material for: INSL3 Variation in Dogs Following Suppression and Recovery of the HPG Axis
Source: Animals (Basel). 2024 Feb 21;14(5):675. doi: 10.3390/ani14050675 (PMC10931505; doi:10.3390/ani14050675)

Supplemental

Supplemental Figure S1

A. Serial dilution of sera from two fetal calves and two male dogs, to indicate assay parallelism. **B**. Titration curve for the Eu^{3+}-labelled tracer against pure canine INSL3 using the heterologous bovine INSL3 assay.

Supplemental Figure S2

Hormone profiles measured in blood samples collected at the indicated times from the cohort of German male Beagle dogs. **A**. Testosterone. **B**. LH. **C**. FSH. For details see [29].

Supplemental Figure S1

A.

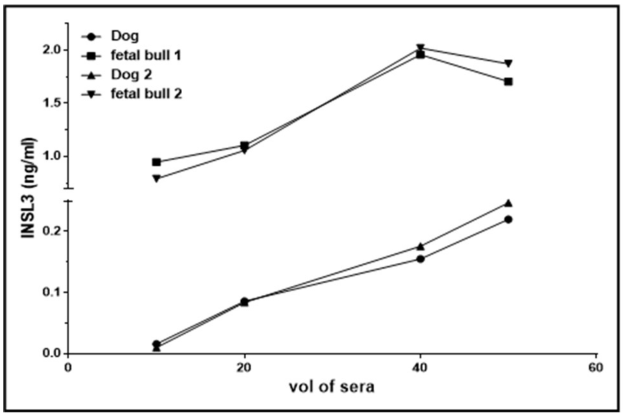

B.

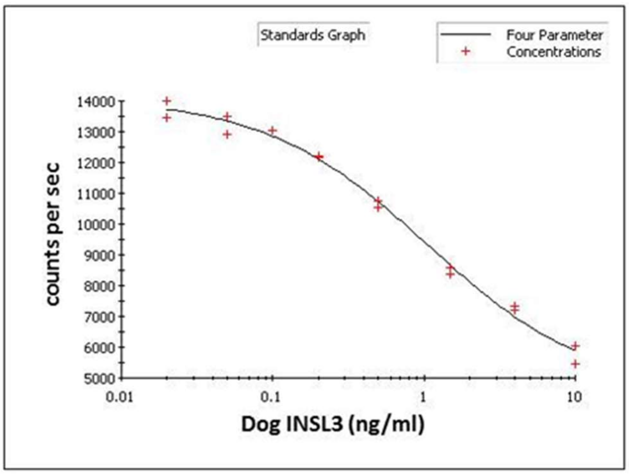

Supplemental Figure S2

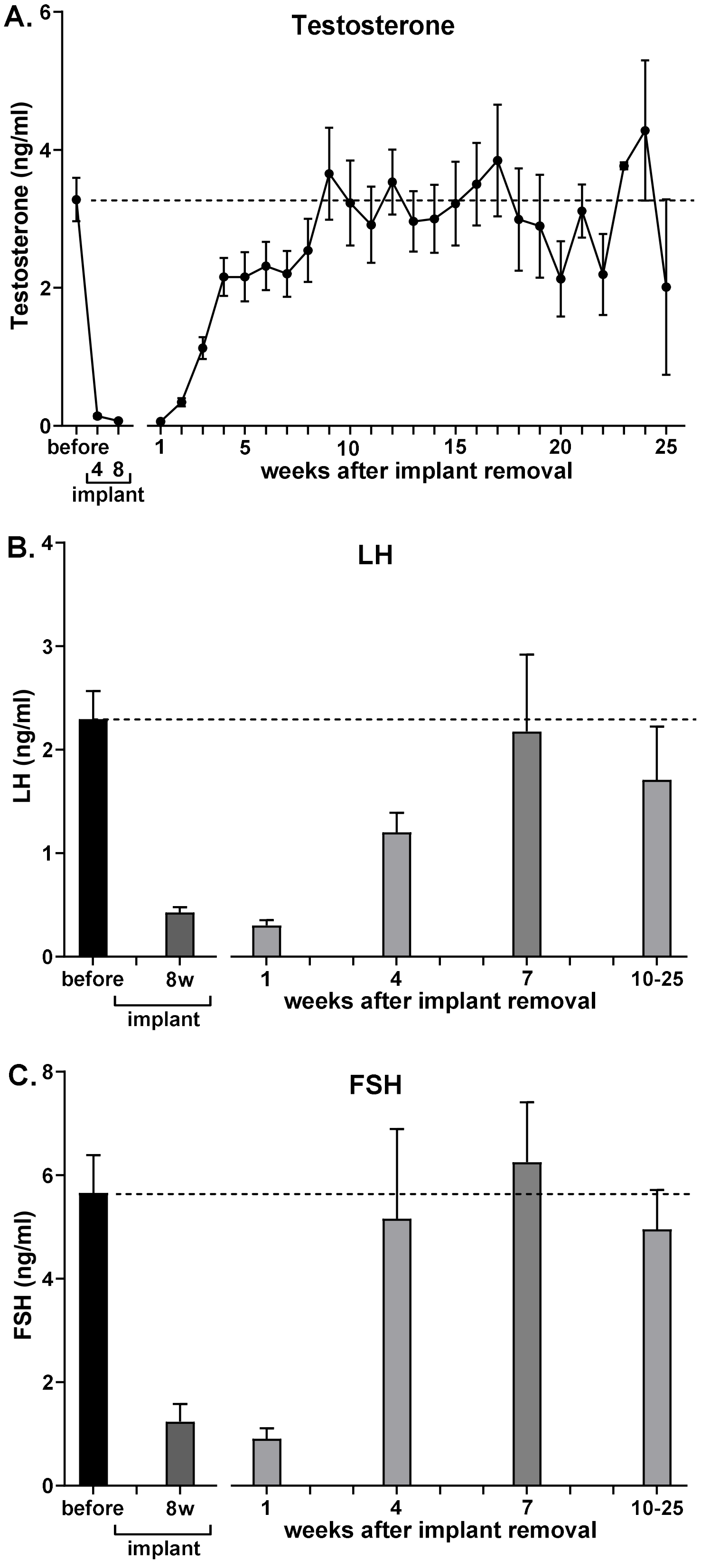

Supplement: Supplementary file 1 [file animals-14-00675-s001.zip › animals-2863064-supplementary.pdf]
